# Rapid Spread and Control of Multidrug-Resistant Gram-Negative Bacteria in COVID-19 Patient Care Units

**DOI:** 10.3201/eid2704.204036

**Published:** 2021-04

**Authors:** Ashka Patel, Michele Emerick, Marie K. Cabunoc, Michelle H. Williams, Michael Anne Preas, Gregory Schrank, Ronald Rabinowitz, Paul Luethy, J. Kristie Johnson, Surbhi Leekha

**Affiliations:** University of Maryland Medical Center, Baltimore, Maryland, USA (A. Patel, M. Emerick, M.K. Cabunoc, M.H. Williams, M.A. Preas);; University of Maryland School of Medicine, Baltimore (G. Schrank, R. Rabinowitz, P. Luethy, J.K. Johnson, S. Leekha)

**Keywords:** COVID-19, coronavirus disease, SARS-CoV-2, severe acute respiratory syndrome coronavirus 2, viruses, respiratory infections, zoonoses, antimicrobial resistance, multidrug resistant, MDR, outbreak, gram-negative, bacteria, bacterial infections, Maryland, United States

## Abstract

We describe rapid spread of multidrug-resistant gram-negative bacteria among patients in dedicated coronavirus disease care units in a hospital in Maryland, USA, during May–June 2020. Critical illness, high antibiotic use, double occupancy of single rooms, and modified infection prevention practices were key contributing factors. Surveillance culturing aided in outbreak recognition and control.

Bacterial colonization and secondary infection have been described in patients hospitalized with coronavirus disease (COVID-19) (*1*,*2*). We report a single-center experience with spread of multidrug-resistant (MDR) gram-negative bacteria (GNB) in COVID-19 patients in Maryland, USA, during May–June 2020. This investigation was determined to be non–human subjects research by the University of Maryland’s Institutional Review Board.

At University of Maryland Medical Center (Baltimore, MD, USA), an 800-bed tertiary-care hospital, since early April 2020, critically ill COVID-19 patients had been housed in 3 dedicated units (*3*), which included 2 intensive care units (ICUs) (units A and B, unit A providing extracorporeal membrane oxygenation support) and 1 intermediate-care unit (unit C). Units were designed as closed, negative-pressure areas where staff remained in the same personal protective equipment while providing care to multiple patients. To accommodate the COVID-19 surge, single-patient ICU rooms in units A and B frequently housed 2 patients. Unit C rooms remained single-occupancy and received patients for step-down care from units A and B. Hospital policy required staff to change gloves and perform hand hygiene (or glove hygiene if wearing 2 layers of gloves) between patients and to wear 2 layers of gowns for patients with resistant organisms and remove the outer gown before moving to the next patient. A team nursing model was used, in which multiple nurses shared responsibilities for each patient during a shift.

For routine surveillance, the hospital defined MDR GNB as Enterobacterales, *Acinetobacter baumannii*, or *Pseudomonas aeruginosa* nonsusceptible to >2 of piperacillin/tazobactam, cefepime, and a carbapenem. Before COVID-19, we performed admission and weekly surveillance for MDR Enterobacterales and *A. baumannii* using perirectal swab specimens on medical and surgical ICU patients and monitored hospitalwide MDR GNB incidence by using the first positive clinical or surveillance culture >48 hours postadmission. 

In mid-May 2020, a cluster of 4 patients with MDR *Escherichia coli* was identified on unit A. Hospitalwide data showed increase in MDR GNB incidence from baseline (Figure, panel A) (weeks 9–11), driven by *E. coli* cases on units A and B (Figure, panel B). Further review also revealed several patients with cefepime-resistant *E. coli* (not meeting institutional MDR criteria), MDR *P. aeruginosa*, and MDR *A. baumannii*. Surveillance screens (perirectal swab specimens on all and sputum on ventilated patients) in the 3 units in week 12 identified 18/29 (62%) additional patients with resistant GNB (MDR GNB, cefepime-resistant *E. coli*, or both). Public health authorities were notified and observations of practice and discussions with leadership were conducted. Twice-weekly surveillance culturing among patients still negative for resistant GNB was instituted (Figure).

**Figure:**
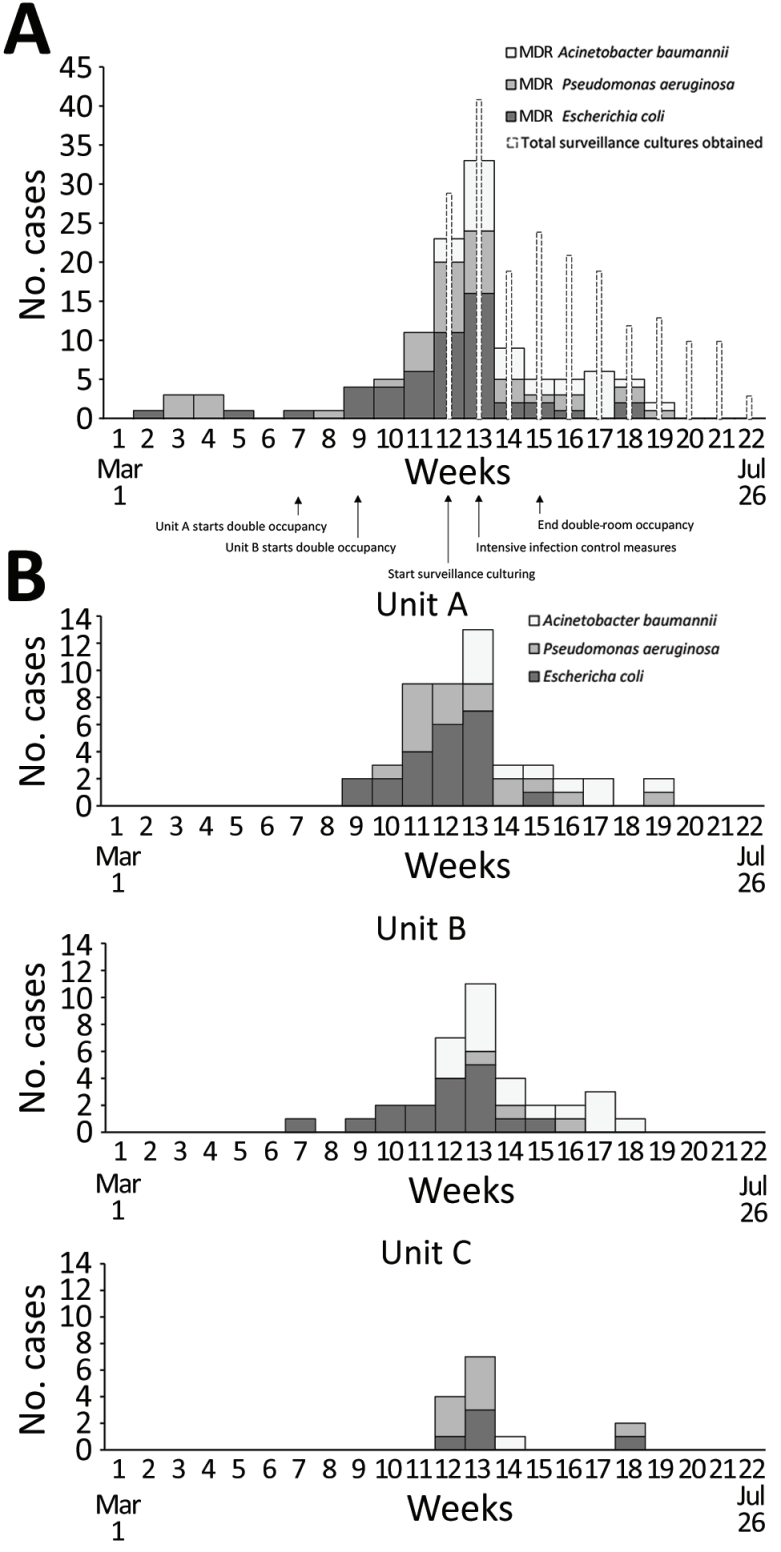
Incidence of patients with a clinical or surveillance culture-positive result indicating MDR or cefepime-resistant *Escherichia coli*, MDR *Acinetobacter baumannii*, or MDR *Pseudomonas aeruginosa* >48 hours after admission to a hospital in Maryland, USA, by week, March 1–July 31, 2020. A) Overall hospitalwide incidence (118 total cases, with 98 positive cultures belonging to outbreak units). Narrow white bars represent the number of surveillance cultures obtained during the outbreak and shaded bars show positive cultures by organism. Arrows show timing of relevant events for transmission and control. B) Incidence of outbreak cases (n = 98) stratified by the 3 units affected by the outbreak. Organisms nonsusceptible to >2 of piperacillin/tazobactam, cefepime, or carbapenem are considered MDR. Patients are included for the first positive culture per organism and therefore might be included more than once. MDR, multidrug-resistant.

During April 16–July 15, a total of 71 unique patients had positive clinical or surveillance cultures for resistant GNB, including 44 *E.coli* (33 MDR and 11 cefepime-resistant), 27 MDR *P. aeruginosa*, and 27 MDR *A. baumannii* (Appendix Table 1). Twenty-four patients (34%) were co-colonized with >1 resistant GNB. Of the 71 patients, 69 (97%) had received antibiotics before first positive resistant GNB culture, 30 (42%) required extracorporeal membrane oxygenation support, 27 (38%) required renal replacement therapy, 52 (73%) received corticosteroids, 25 (35%) received remdesivir, and 14 (20%) received tocilizumab. Twenty-three (32%) patients ultimately died.

Relatedness of early *E. coli* isolates was assessed by pulsed-field gel electrophoresis (PFGE) (n = 13, weeks 7–11) and genetic β-lactamase determination by Verigene gram-negative blood culture nucleic acid test (Luminex Corporation, https://www.luminexcorp.com) (n = 38, weeks 7–14) (*4*; Appendix). PFGE revealed 3 groups. Groups 1 and 2 (n = 7) were considered related and were negative for β-lactamases; these and 8/10 additional β-lactamase-negative isolates were from unit B. Group 3 (n = 6) isolates did not produce bands but were positive for CTX-M; these and 14/15 additional CTX-M positive isolates (including 10/11 phenotypically cefepime-resistant but not MDR) were from unit A and considered related, suggesting rapid patient-to-patient transmission (Appendix Table 1). MDR *P. aeruginosa* transmission occurred predominantly in unit A, whereas MDR *A. baumannii* was largely in unit B. Resistant GNB were likely introduced into unit C from both units A and B (Figure, panel B).

Key infection control findings (*5*) included tight physical spaces and close proximity of patients in double occupancy (*6*), multiple staff in contact with each patient in the team nursing model, and low compliance with hand and glove hygiene and gown changes between patients. To limit staff exposure to COVID-19 patients, the unit had less support from ancillary services; instead, daily room and equipment cleaning and stocking of medications and supplies were performed by unit-based clinical staff.

Outbreak control interventions included discontinuation of double occupancy, frequent infection prevention rounds to promote hand hygiene and glove and gown changes between patients, increased environmental services support, and attention to disinfection of reusable equipment and high-touch surfaces (Appendix Table 2) (*7*). Surveillance culturing showed a decrease in positive cultures over time (Figure).

Prolonged critical illness, high antibiotic and corticosteroid use, double occupancy, the team nursing model, and modified infection prevention practice were considered contributors to transmission, underscoring the importance of vigilance to MDR organisms in this setting (*5*,*7*–*10*). Surveillance culturing aided with recognizing the extent of spread and informed early intervention.

Appendix Additional information about rapid spread and control of multidrug-resistant gram-negative bacteria in COVID-19 patient care units.
