# Supplementary material for: Rapid Spread and Control of Multidrug-Resistant Gram-Negative Bacteria in COVID-19 Patient Care Units
Source: Emerg Infect Dis. 2021 Apr;27(4):1234–7. doi: 10.3201/eid2704.204036 (PMC8007317; doi:10.3201/eid2704.204036)
Supplement: Appendix — Additional information about rapid spread and control of multidrug-resistant gram-negative bacteria in COVID-19 patient care units. [file 20-4036-Techapp-s1.pdf]

Article DOI: https://doi.org/10.3201/eid2704.204036

Rapid Spread and Control of Multidrug-Resistant Gram-Negative Bacteria in COVID-19 Patient Care Units

Appendix

Microbiologic and Molecular Analysis

Definitions of Resistant Gram-Negative Bacteria Based on Antimicrobial-Susceptibility Testing

At our institution, we define multidrug-resistant Gram-negative bacteria (MDR)-GNB as Enterobacterales, *Acinetobacter baumannii*, or *Pseudomonas aeruginosa* non-susceptible (intermediate or resistant) to $\geq$2 of the following: piperacillin-tazobactam, cefepime, and any carbapenem (carbapenem testing includes meropenem and/or imipemen for *P. aeruginosa* and *A. baumannii*, and ertapenem and meropenem for Enterobacterales and non-susceptible to only one is required to meet the MDR definition). In addition to MDR-GNB as defined, the outbreak also included several isolates of *E.coli* that were cefepime-resistant but did not meet the institutional definition of MDR. These are collectively referred to as "resistant-GNB" for purposes of the outbreak.

Strain Characterization by Pulsed-Field Gel Electrophoresis

To determine the genetic relatedness of *E. coli* isolates from the outbreak analyzed in this study, 13 isolates were sub-cultured to agar slants and sent to ARUP Laboratories (Salt Lake City, UT) for bacterial strain characterization by Pulsed Field Gel Electrophoresis (PFGE). Genetic relatedness was determined by comparing the DNA band pattern within the agar gel. Varying levels of relatedness were assigned based on the number of differences between DNA bands. Specifically, ARUP Laboratories recommends the following non-standardized criteria using the numbers of band differences to aid interpretation in conjunction with epidemiologic information: 0 – indistinguishable, part of the outbreak; 2–3 – closely related, probably part of the outbreak; 4–6 – possibly part of the outbreak; and ≥7 – not part of the outbreak. Based on these results, early outbreak isolates were assigned into PFGE groups 1, 2, and 3. Isolates within

group 1 (n = 2) were considered indistinguishable from each other and isolates within group 2 (n = 5) were considered indistinguishable from one another; groups 1 and 2 differed by 2 bands and were considered closely related. Group 3 (n = 7) failed to produce bands and could not be analyzed by this method.

Detection of Antimicrobial-Resistance Genes

The Verigene Gram-negative blood culture nucleic acid test (BC-GN, Luminex Corporation, Austin, TX) was used to determine whether 31 *E. coli* isolates grown from outbreak patients were carrying a common resistance mechanism. The nucleic acid test detects six resistance markers: CTX-M, KPC, NDM, VIM, IMP, and OXA. Carriage of a resistance mechanism between isolates with common antimicrobial susceptibility and genetic patterns may mean the organisms are epidemiologically related. Although the nucleic acid test is meant for blood cultures, it can also be used with isolates following a procedure provided by the manufacturer. Briefly, a 0.5 McFarland dilution of the *E. coli* isolate in question was created in sterile saline. 700 µL of this solution was then pipetted into the sample well of the test cartridge and the test was run following the company’s instructions per the package insert. Following bacterial DNA extraction, the DNA is hybridized to target-specific capture DNA located on a microarray, further hybridized to gold nanoparticles, and enhanced with silver particles to allow for target detection by an optical reader. Detection of each nucleic acid target is reported through Verigene software.

Appendix Table 1. Antimicrobial-susceptibility testing results of *Acinetobacter baumannii*, *Escherichia coli*, and *Pseudomonas aeruginosa* isolates recovered from outbreak specimens and genetic relatedness as determined by pulsed-field gel electrophoresis and presence of antimicrobial resistance genes in *E. coli* isolates*

Unit	Specimen Source of First Positive Culture	Week First Detected	Organism	Pip/Tazo	Cefepime	Carbapenem	PFGE Group	Beta-lactamase detection by Verigene BC-GN
B	Sputum	7	EC	R	R	R	1	Not detected
A	Sputum	9	EC	R	R	I	3	CTX-M
A	Bronchial	9	EC	R	R	S	3	CTX-M
B	Sputum	10	EC	R	R	S	2	Not detected
B	Sputum	10	EC	R	R	R	2	Not detected
A	Sputum	10	EC	R	R	S	3	CTX-M
B	Sputum	10	EC	R	R	R	2	Not detected
A	Bronchial	10	EC	R	R	S	3	CTX-M
B	Sputum	11	EC	R	R	R	2	Not detected
B	Sputum	11	EC	R	R	R	1	Not detected
B	Sputum	11	EC	R	R	R	2	Not detected
A	Sputum	11	EC	S	R	S	3	CTX-M
A	Sputum	11	EC	S	I	S	3	CTX-M
A	Sputum	11	EC	S	R	S		CTX-M
A	Sputum	12	EC	S	R	S		CTX-M
A	Rectal	12	EC	R	R	S		CTX-M
A	Sputum and rectal	12	EC	S	R	S		CTX-M
A	Sputum and rectal	12	EC	S	R	S		CTX-M
A	Sputum and rectal	12	EC	R	R	S		CTX-M
A	Rectal	12	EC	R	R	S		CTX-M
B	Sputum and rectal	12	EC	R	R	S		Not detected
B	Sputum and rectal	12	EC	S	R	S		Not detected
B	Rectal	12	EC	R	R	R		Not detected
B	Rectal	12	EC	R	R	R		Not detected
C	Urine	12	EC	R	R	S		Not detected
A	Sputum and rectal	13	EC	S	R	S		CTX-M
A	Sputum	13	EC	S	R	S		CTX-M
C	Rectal	13	EC	R	R	R		
B	Rectal	13	EC	R	R	R		Not detected
B	Sputum	13	EC	R	R	R		
A	Sputum	13	EC	R	R	S		
C	Rectal	13	EC	S	R	S		CTX-M
A	Blood	13	EC	S	R	S		CTX-M
A	Sputum	13	EC	I	R	S		CTX-M
A	Sputum and rectal	13	EC	I	R	S		CTX-M
A	Sputum	13	EC	R	R	S		CTX-M
A	Sputum	13	EC	S	R	S		CTX-M
B	Sputum	13	EC	R	R	S		Not detected
B	Blood	13	EC	R	R	S		Not detected
A	Rectal	13	EC	R	R	S		Not detected
B	Sputum	14	EC	R	R	S		Not detected
A	Rectal	15	EC	I	R	S		
B	Sputum	15	EC	R	R	R		
C	Rectal	18	EC	R	I	S		
A	Sputum	10	PA	I	I	S		
A	Sputum	11	PA	I	I	S		
A	Sputum	11	PA	I	NT	R		
A	Sputum	11	PA	I	I	R		
A	Sputum	11	PA	R	I	S		
A	Sputum	11	PA	S	I	R		
C	Sputum	12	PA	I	I	S		
A	Sputum	12	PA	I	I	R		
A	Sputum	12	PA	I	I	S		
A	Sputum	12	PA	I	S	I		
C	Sputum	12	PA	I	R	R		
C	Urine	12	PA	R	R	S		
C	Sputum	13	PA	R	R	R		
C	Sputum	13	PA	I	I	R		
C	Rectal	13	PA	I	R	S		
B	Sputum and rectal	13	PA	R	R	R		
A	Bronchial	13	PA	I	S	R		
A	Sputum	13	PA	R	S	R		
C	Sputum	13	PA	R	R	R		
A	Sputum	14	PA	I	I	R		

Unit	Specimen Source of First Positive Culture	Week First Detected	Organism	Pip/Tazo	Cefepime	Carbapenem	PFGE Group	Beta-lactamase detection by Verigene BC-GN
A	Sputum	14	PA	I	S	R		
B	Sputum	14	PA	I	S	R		
A	Sputum	15	PA	I	S	R		
A	Sputum	16	PA	S	R	R		
B	Sputum and rectal	16	PA	I	S	R		
C	Rectal	18	PA	R	I	R		
A	Sputum	19	PA	I	I	S		
B	Sputum	12	AB	R	R	R		
B	Blood	12	AB	R	R	R		
B	Sputum and rectal	12	AB	R	R	R		
B	Sputum	13	AB	R	R	R		
B	Rectal	13	AB	R	R	R		
B	Sputum	13	AB	R	R	R		
A	Sputum	13	AB	R	R	R		
B	Sputum	13	AB	R	R	R		
B	Sputum	13	AB	R	R	R		
A	Sputum	13	AB	R	R	R		
A	Sputum	13	AB	R	R	R		
A	Blood	13	AB	R	R	R		
B	Rectal	14	AB	R	R	R		
B	Rectal	14	AB	R	R	R		
C	Rectal	14	AB	R	R	R		
A	Sputum	14	AB	R	I	R		
B	Bronchial	15	AB	R	R	R		
A	Sputum	15	AB	R	R	R		
A	Sputum	16	AB	R	I	R		
B	Sputum and rectal	16	AB	R	R	R		
B	Sputum	17	AB	R	R	R		
A	Sputum	17	AB	R	R	R		
A	Sputum	17	AB	R	R	R		
B	Sputum	17	AB	R	NT	R		

*Bacterial isolates of *Acinetobacter baumanii* (AB), *Escherichia coli* (EC), and *Pseudomonas aeruginosa* (PA) that were isolated from patient clinical and surveillance specimens are listed, along with the hospital unit, week first isolated, and culture specimen source (n = 98; 44 EC, 27 PA and 27 AB). The list includes multiple isolates from the same patient, if co-colonized. The antimicrobial susceptibility testing pattern for piperacillin/tazobactam (Pip/Tazo), cefepime, and the carbapenems (meropenem and/or imipemen for *P. aeruginosa* and *A. baumannii*, and ertapenem and/or meropenem for *E. coli*) is also listed for each isolate as sensitive (S), intermediate (I), resistant (R), or not tested (NT). In addition, for *E. coli* outbreak isolates, pulsed field gel electrophoresis (PFGE) was used to determine genetic relatedness of 13 early *E. coli* isolates, and their corresponding genetic grouping is shown. Furthermore, the Verigene Gram-negative blood culture nucleic acid test (Verigene BC-GN) was performed to determine the presence of antimicrobial resistance markers in 38 of the *E. coli* isolates. Of the six β-lactamase resistance genetic markers on the nucleic acid test, only CTX-M was detected, and the presence of CTX-M or absence of β-lactamases in each tested isolate is detailed.

Appendix Table 2. Infection prevention and control observations and measures of compliance pre-COVID-19 baseline, during outbreak, and following institution of outbreak control interventions

Domain	Pre-COVID baseline	During-outbreak	During and post-intervention
Hand hygiene or glove hygiene	• Routine hand hygiene practice; single pair of gloves, if worn, routinely changed between patients<br><br>• Compliance for 2 quarters (October 2019-March 2020) 81% - 99% from anonymous observer hand hygiene monitoring program data (n ≥ 30 observations per unit per month)	• One or two layers of gloves continuously worn<br><br>• Most commonly practiced glove decontamination without change of gloves<br><br>• Not formally measured but low self-reported compliance particularly when moving between two patients in the same ICU room	• Practiced double gloving, removal of outer layer with glove hygiene between two patients<br><br>• Self-reported to be higher<br><br>• Formally measured glove hygiene compliance for Unit A 100% (n = 9)
Glove and gown change practice	• Gloves and gowns routinely removed following each patient encounter	• Not changed between patients, base gown and gloves worn continuously for multiple patient encounters in COVID-19 patient care unit	• Double gowning for MDR organism rooms, double glove with removal of outer layer of gloves and gowns upon exit and glove hygiene
Management of shared equipment and supplies	• Adequate space for supplies<br><br>• Shared equipment e.g., beds, dialysis machines, IV pumps and feeding pumps, routinely returned to central equipment distribution for thorough cleaning and disinfection	• Lack of storage space for supplies; stored on countertops and basins precluding adequate disinfection of surfaces<br><br>• Most equipment remained on unit for disinfection between patients	• Dedicated supplies storage space created to allow better disinfection of horizontal surfaces<br><br>• Resumed return of equipment to central equipment distribution for thorough cleaning and disinfection
Environmental services support	• Regular support<br><br>• Daily and terminal cleaning of all rooms by EVS	• Limited support<br><br>• Unit-based patient care staff responsible for cleaning inside unit; EVS did not routinely enter unit except for terminal cleaning upon request	• Enhanced support<br><br>• EVS staff assigned for daily and terminal cleaning
Compliance with disinfection of high-touch surfaces and shared equipment	• Compliance not formally measured	• Compliance with high-touch surface and shared equipment measured using fluorescent gel removal: Unit A 23/27 (85%); Unit B 9/14 (64%)	• Compliance with high-touch surface and shared equipment measured using fluorescent gel removal: Unit A 75/80 (91%); Unit B 54/70 (77%)
Double occupancy of single rooms	• None/not applicable	• 40%–50% on average, peaked in weeks 10–13	• Declined to none by week 15
